# Cardiac adaptation in pediatric kidney transplant recipients with large donor–recipient size discrepancy: a speckle-tracking echocardiography study

**DOI:** 10.1007/s00467-026-07368-6

**Published:** 2026-06-02

**Authors:** Mark W. Schaars, Floris E. A. Udink ten Cate, Elisabeth A. M. Cornelissen, Gert Weijers, Marieke Voet, Anne E. C. M. Saris

**Affiliations:** 1 Technical Medicine, University of Twente, Enschede, The Netherlands; 2 Medical Ultrasound Imaging Centre, Department of Medical Imaging, Radboud University Medical Centre, Nijmegen, the Netherlands; 3 Department of Pediatric Cardiology, Amalia Children’s Hospital, Radboud University Medical Center, Nijmegen, The Netherlands; 4 Department of Pediatric Nephrology, Amalia Children’s Hospital, Radboud University Medical Center, Nijmegen, The Netherlands; 5 Department of Anesthesiology, Pain and Palliative Care, Radboud University Medical Center, Nijmegen, the Netherlands

**Keywords:** Speckle-tracking echocardiography, Global longitudinal strain, Kidney transplantation, Donor-recipient size mismatch, Pediatric

## Abstract

**Background:**

Pediatric living donor kidney transplantation (KT) with large donor-recipient size mismatch induces a large hemodynamic burden on the child recipient. Although successful KT is known to be beneficial for cardiac function and remodeling, its effects in small recipients with adult donors have not been evaluated. We aimed to describe left ventricular (LV) function and geometry using conventional and speckle-tracking echocardiography in this population.

**Methods:**

A prospective longitudinal study was performed. Living donor KT recipients with large donor-recipient size mismatch underwent conventional and speckle-tracking echocardiography prior to KT, shortly after KT and at 6 and 12 months post-transplantation. LV global longitudinal strain (GLS), LV ejection fraction, cardiac index, LV mass index (LVMi) and LV dimensions were obtained.

**Results:**

Ten patients were included with a median [IQR] age of 5.9 years [4.4–8.5]. The median donor–recipient body surface area ratio was 2.36. Prior to transplantation, LV systolic dysfunction was found in 80% of the patients (GLS −18.9% [−19.7–−18.7]). Significant improvement was found after 12 months (change in GLS −1.3% [−1.8–−0.9], *p* = 0.004). A significant reduction in LVMi was found at 6 months and 12 months of −8.9 g/m^2.7^ [−15.6–−5.4], *p* = 0.01 and −14.3 g/m^2.7^ [−15.3–−12.9], *p* = 0.004, respectively. No significant changes in cardiac index were found at 6 and 12 months.

**Conclusions:**

Despite the increased hemodynamic demands after pediatric KT with large donor-recipient size mismatch, improved LV function and reduced left ventricular mass were found within 1 year after transplantation. These results suggest reversed cardiac remodeling.

**Graphical abstract:**

**Figure:**
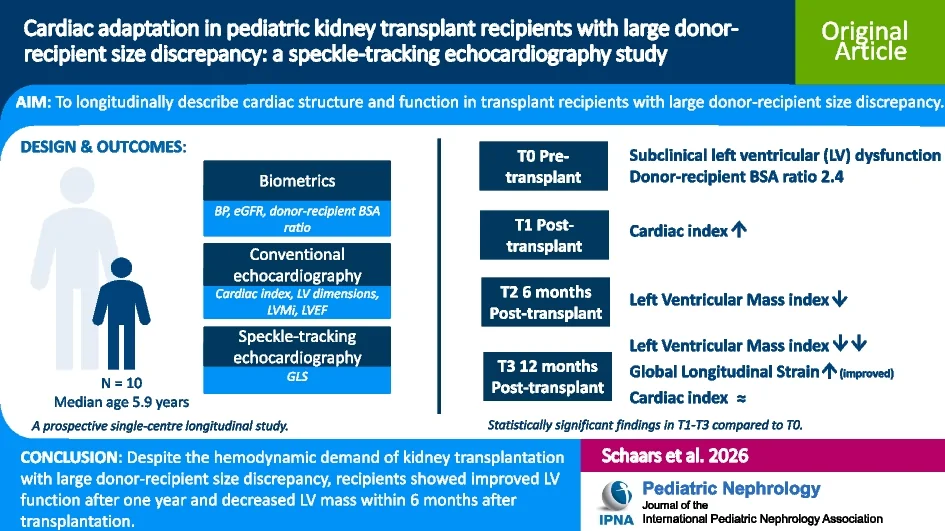
A higher resolution version of the Graphical abstract is available as Supplementary information

**Supplementary Information:**

The online version contains supplementary material available at https://doi.org/10.1007/s00467-026-07368-6.

## Introduction

Kidney failure in children, as in adults, burdens the cardiovascular system as is reflected by adverse cardiac remodeling and increased cardiovascular disease-related mortality [1, 2]. Deterioration of cardiovascular function continues during hemodialysis, which itself poses strain on the heart and constitutes an independent cardiovascular risk factor [3]. Kidney transplantation (KT) is the preferred treatment and, when successful, alleviates cardiorenal stressors. Although KT with a living donor grants the best nephrologic outcome [4], it introduces unique complexities due to the large donor–recipient size discrepancy in young recipients. Surgically, vessel size mismatch and intra-abdominal placement can be challenging. Hemodynamically, a relatively large organ with low vascular resistance is introduced into the child’s circulation, effectively functioning as a large arteriovenous shunt. The adult donor kidney demands a substantial blood flow, imposing a disproportionately high hemodynamic demand on a small recipient [5]. During the perioperative period, advanced hemodynamic monitoring and support is needed to meet this demand. However, the long-term cardiac response to this increased cardiac workload is not yet investigated. Kidney transplantation has been shown to have beneficial effects on cardiac remodeling as illustrated by echocardiographic findings [6, 7]. However, it is unknown whether these beneficial adaptations occur in pediatric recipients of an adult donor kidney with significant donor–recipient size mismatch.

Traditionally, cardiac function, and specifically left ventricle (LV) function is described using conventional echocardiograph parameters such as left ventricular ejection fraction (LVEF). Speckle-tracking echocardiography (STE) is an advanced imaging technique that enables precise analysis of cardiac tissue motion. Speckles are natural acoustic markers, unique to a small region of tissue that remain relatively stable during motion, allowing them to be tracked over time to estimate local tissue displacement. STE allows quantitative assessment of local myocardial deformation during the cardiac cycle. Longitudinal, i.e., apicobasal, left ventricle deformation is described by the global longitudinal strain (GLS), in which systolic shortening is indicated as a negative percentage value. GLS is a reproducible and robust parameter and has been shown to be more sensitive in detecting subclinical myocardial dysfunction than LVEF [8]. STE and GLS have demonstrated value in identifying early myocardial dysfunction in the context of kidney failure and hemodialysis [1, 3].

This study aims to describe the longitudinal adaptation of cardiac function and remodeling after pediatric kidney transplantation with large donor–recipient size mismatch, using conventional echocardiography and STE up to 1 year post-transplantation.

## Methods

### Study population

Participants were selected from the Circulation and Hemodynamics in Living Donor Kidney Transplantation in Children (CHILD-KiTC) trial (Dutch Trial Register NL6666). The CHILD-KiTC study is a prospective, ongoing, single center, observational study investigating the hemodynamic changes occurring in living donor KT with large donor–recipient size-mismatch. Inclusion criteria are under 15 years of age and body weight <40 kg. Patients with congenital heart diseases were excluded. The study received approval of the institutional medical ethical committee (METC Oost-Nederland, approval number NL61392.091.17). All participants gave their written informed consent to participate in this study which was carried out in compliance with the principles of the declaration of Helsinki. For this substudy, all participants who completed the full echocardiography series according to study protocol were selected for analysis.

### Procedure

All patients underwent living donor KT according to a local protocolized multidisciplinary program. In children with a bodyweight <20 kg, the graft was placed intra-abdominally. Vascular anastomoses were made on the abdominal aorta and inferior vena cava, by discretion of the surgeon. Immunosuppressive regimen consisted of induction with basiliximab and the TWIST protocol thereafter [9].

### Data collection

Patient characteristics gathered included age, sex, length, weight, co-morbidities, blood pressure, presence of dialysis prior to KT, etiology of kidney failure and use of antihypertensive medication. Both recipient and donor body surface area (BSA) (Dubois formula) were gathered and the BSA ratio between donor and recipient was calculated. BMI Z-scores based on gender and age were computed using WHO Child Growth Standards. The glomerular filtration rate (eGFR) was calculated using serum creatinine according to the CKiD-U25 formula [10].

### Echocardiographic protocol

Transthoracic echocardiography examinations were performed within 3 months before transplantation (T0), after transplantation at the pediatric intensive care unit (PICU) (T1) and at 6 months (T2) and 12 months (T3) after transplantation in an outpatient setting. Patients were positioned in left lateral decubitus position when feasible. At the PICU, this was typically not achievable and examinations were conducted in supine position.

All images were acquired by an experienced pediatric sonographer or pediatric cardiologist using a GE ultrasound machine with appropriate pediatric cardiac probe (GE Vingmed Ultrasound, Horten, Norway). LV dimensions, volumes, fractional shortening (FS), LVEF, left ventricle mass (LVM) and relative wall thickness (RWT) were measured or calculated according to the American Society of Echocardiography (ASE) guidelines [11]. LV dimensions were acquired from M-mode images acquired in the parasternal long axis view. LVM was calculated according to the Devereux formula and allometrically indexed to height to the power of 2.7 [12]. Cardiac output was calculated using the left ventricle outflow tract (LVOT) pulsed wave Doppler velocity time integral, LVOT diameter and heart rate. Cardiac output was divided by BSA to find the cardiac index (CI). BSA-adjusted Z-scores were computed for LV dimensions according to Pettersen et al. [13]. All image analyses, including STE measurements, took place offline using EchoPAC software (version 206, GE Healthcare) at a dedicated workstation. Image analysis was performed by a trained investigator (MS, technical physician) with training and supervision provided by a pediatric cardiologist (FU).

### Speckle-tracking echocardiography

2D grayscale standard apical long axis, two chamber and four chamber view recordings were used for strain analysis in EchoPAC’s Automated Functional Imaging (AFI) module. In each view, the myocardium was delineated manually such that the region of interest encompassed the entire myocardium. Automated speckle-tracking by the software followed. Tracking was visually inspected and the region of interest was redefined when necessary. Each apical view is automatically divided into six standard segments, resulting in a total of 18 segments. Longitudinal end-systolic strain of each of the 18 segments was averaged to find global longitudinal strain (GLS). Based on visual inspection and only after cross-checking with a second observer, a maximum of 2 out of 18 segments were excluded in the GLS calculation in cases of poor tracking. End systole was defined by aortic valve closure as observed in the pulsed wave Doppler signal of the LV outflow tract in accordance with ASE guidelines [14]. Three cardiac cycles with similar heartrate were selected per view; individual cycles were reused when fewer than three cycles of sufficient image quality were available. The GLS was obtained by averaging over these three cycles.

For healthy children aged 2 to 9 years, Levy et al.’s meta-analysis reported a mean GLS of −21.7% (95% CI −23.0% to −20.5%) [15]. In the present study, the lower limit of −20.5% was considered the threshold for impaired global longitudinal strain for all patients.

### Statistical analysis

Statistical analysis was performed in consultation with a senior statistician. Normality testing was not feasible given the limited sample size (*n* = 10) and because of the absence of prior evidence regarding distribution, normality was not assumed. Consequently, non-parametric methods were applied and data are presented as median values with interquartile ranges. For comparison between baseline (T0) and follow-up (T1, T2 and T3) the two-tailed paired Wilcoxon signed-rank test was used. Statistical significance was defined as *p* < 0.05. All statistical analyses were performed using MATLAB 2023a (Mathworks Inc, Natick, MA, USA).

## Results

### Study population

Complete echocardiography datasets were obtained from 10 participants of the CHILD-KiTC study. Patient characteristics before transplantation are summarized in Table 1. Median age of our patients was 5.9 years. The etiology of kidney failure was congenital nephrotic syndrome for 5 children (50%), obstructive uropathy for 4 children (40%), polycystic kidneys for 1 child (10%) and kidney dysplasia due to VACTERL association for 1 child (10%). Two patients received hemodialysis and 5 patients received peritoneal dialysis prior to kidney transplantation. Preemptive transplantation was performed in 3 patients. Use of antihypertensive medication, blood pressure and eGFR measurements at time of each echocardiography exam are summarized in Table 2.

**Table 1:** Patient characteristics before transplantation

	Patients (*n* = 10)
Age (years)	5.9 [4.4–8.5]
Gender (male/female)	7/3
Weight (kg)	20.5 [14.5–23.6]
Height (cm)	112 [96–122]
BMI	15.8 [15.1–16.5]
BMI (Z-score)	0.45 [−0.10–0.73]
BSA (m^2^)	0.79 [0.60–0.91]
D/R BSA ratio	2.36 [2.08–2.76]
Systolic blood pressure (mmHg)	118 [110–123]
Diastolic blood pressure (mmHg)	74 [59–77]
Patients on antihypertensive medication	7 (70%)
ACE inhibitor	3 (30%)
CCB	3 (30%)
CCB and beta blocker	1 (10%)
Heartrate (bpm)	91 [86–110]
eGFR (ml/min/1.73 m^2^)	4 [3–6]

All values are expressed as median [IQR] or as percentage

*BMI* body mass index, *BSA* body surface area, *D/R BSA ratio* donor/recipient BSA ratio, *ACE inhibitor* angiotensin-converting enzyme inhibitors, *CCB* calcium channel blocker, *eGFR* estimated glomerular filtration rate (CKiD-U25 formula)

**Table 2:** Patient characteristics at predefined echocardiography exams among 10 patients

	T0 (Pre-transplant)	T1 (PICU)	T2 (6 months)	T3 (12 months)	*p* T0 vs. T1	*p* T0 vs. T2	*p* T0 vs. T3
Systolic blood pressure (mmHg)	118 [110–123]	123 [104–136]	110 [101–114]	106 [99–113]	*0.363*	*0.094*	***0.043***
Diastolic blood pressure (mmHg)	74 [59–77]	74 [62–98]	65 [56–74]	59 [57–66]	*0.152*	*0.139*	*0.055*
Patients on antihypertensive medication	7 (70%)	-	3 (30%)	3 (30%)	*-*	*-*	*-*
ACE inhibitor	3 (30%)	-	1 (10%)	1 (10%)	*-*	*-*	*-*
CCB	3 (30%)	-	1 (10%)	1 (10%)	*-*	*-*	*-*
Beta blocker	-	-	1 (10%)	1 (10%)	*-*	*-*	*-*
CCB and beta blocker	1 (10%)	-	-	-	*-*	*-*	*-*
eGFR (ml/min/1.73 m^2^)	5 [3–8]	58 [48–97]	75 [59–90]	63 [53–74]	***0.002***	***0.002***	***0.002***

Values are expressed as median with interquartile ranges or as numbers of patients with percentage. For comparison of T0 to T1, T2 and T3, the paired Wilcoxon test was used. Significant p-scores are in bold

*ACE inhibitor* angiotensin-converting enzyme inhibitors, *CCB* calcium channel blocker, *eGFR* estimated glomerular filtration rate (CKiD-U25 formula)

### Echocardiographic parameters

**Fig. 1:**
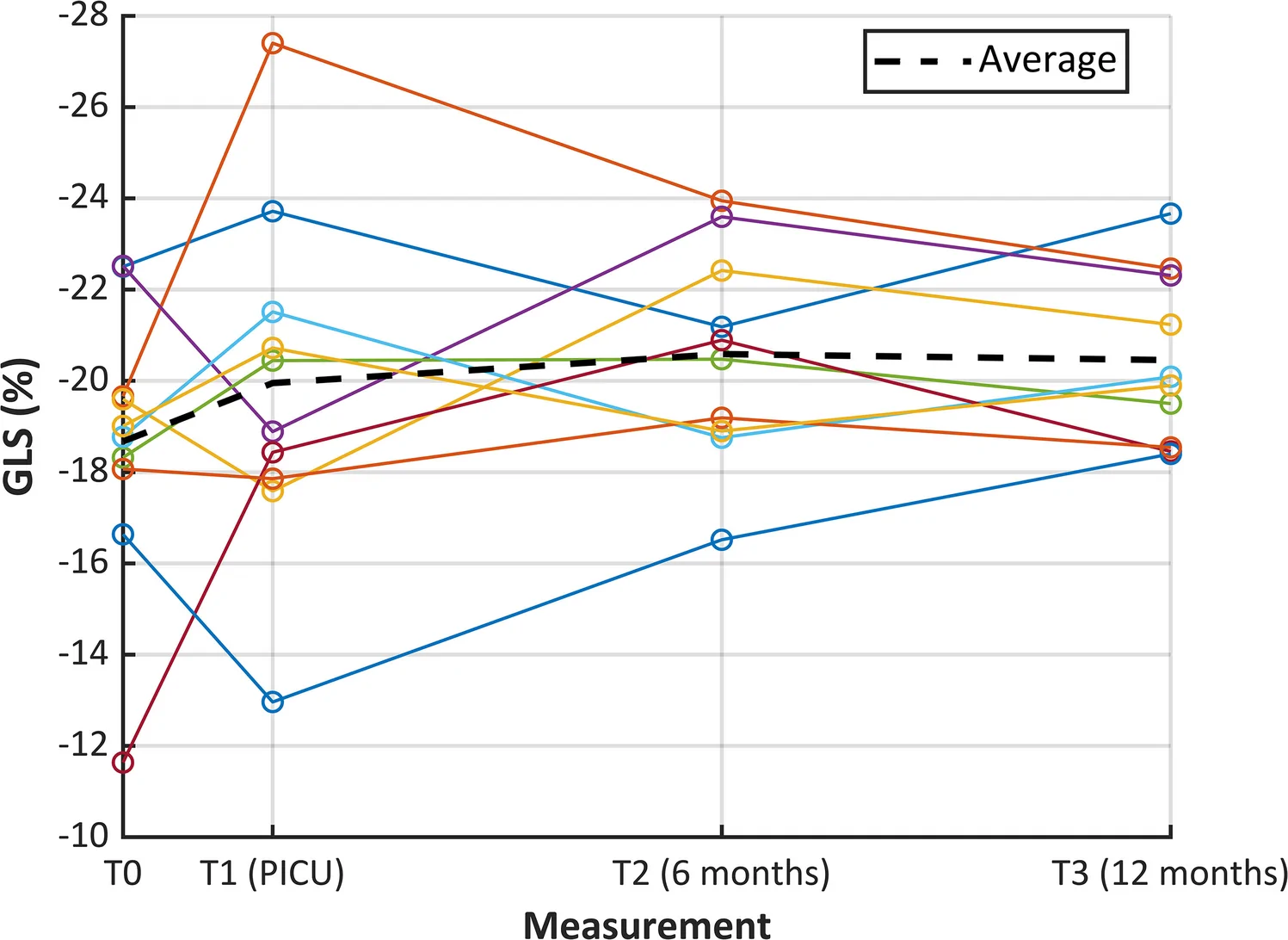
Global longitudinal strain values across four measurement time points. Colored lines represent individual patients’ values. Average GLS values are indicated by the dashed line. Y-axis is inverted

**Fig. 2:**
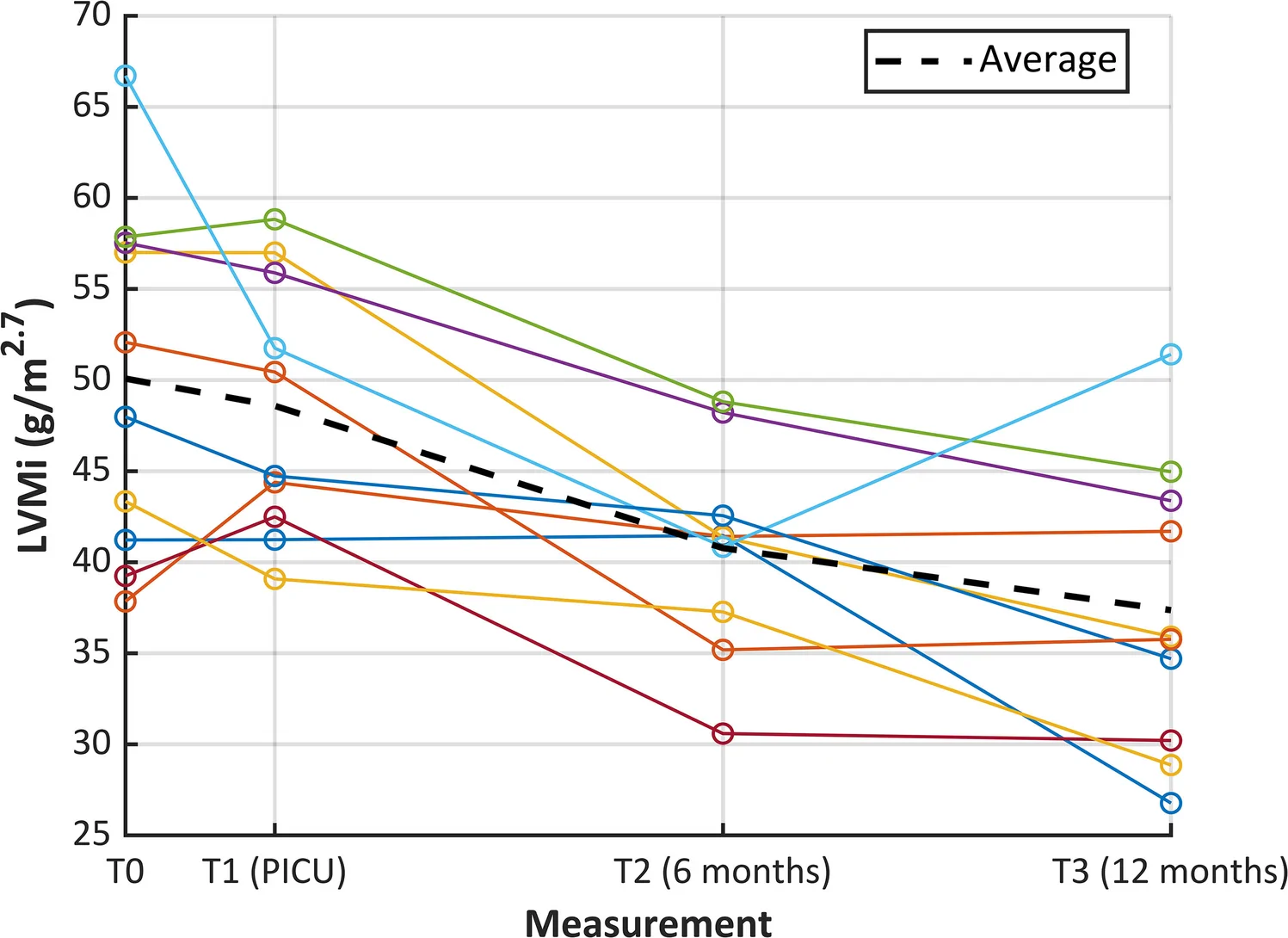
Left ventricular mass index values across four measurement time points. Colored lines represent individual patients’ values. Average LVMi values are indicated by the dashed line

Table 3 summarizes the echocardiographic parameters on LV structure and function. Patient individual trends for GLS and LVMi are shown in Figs. 1 and 2, respectively. Shortly after transplantation at the PICU (T1), 6/10 (60%) children showed a decrease in GLS, indicating improved left ventricular systolic function. After 6 months, 6/10 (60%) children showed a decrease in GLS. After 12 months, 9/10 (90%) showed a decrease in GLS with a mean change in GLS of −1.8%. At T0, 8/10 (80%) patients showed impaired function based on GLS (GLS > −20.5%). After 6 months, 5/10 patients showed impaired GLS and after 12 months, 6/10 did. After 12 months (T3), 9/10 (90%) children showed a significantly decreased LVMi with a mean decrease of 12.7 g/m^2.7^.

**Table 3:** Conventional and speckle-tracking echocardiographic measurements among 10 patients

	T0 (Pre-transplant)	T1 (PICU)	T2 (6 months)	T3 (12 months)	*p* T0 vs. T1	*p* T0 vs. T2	*p* T0 vs. T3
CI [ml/min/m^2^]	3868 [3577–4922]	5847 [5080–6075]	4328 [3656–5156]	3719 [3230–5534]	***0.006***	*0.375*	*1*
LV EF (%)	49.90 [47.24–50.86]	52.79 [49.88–58.26]	53.06 [51.58–55.87]	51.93 [49.35–54.89]	*0.105*	***0.049***	***0.027***
FS (%)	40 [37–41]	49 [39–52]	38 [36–41]	40 [34–45]	*0.131*	*0.922*	*0.77*
Z-score IVSd	0.65 [0.18–0.84]	0.89 [0.05–1.60]	0.54 [−0.21–0.90]	0.08 [−0.24–0.28]	*0.232*	*0.432*	*0.193*
Z-score LVPWd	2.43 [2.09–3.08]	2.40 [1.70–3.69]	1.79 [1.55–2.49]	1.85 [1.13–2.11]	*1*	***0.037***	***0.049***
Z-score LVIDd	0.22 [−1.69–0.69]	−1.23 [−1.46 to −0.17]	−0.80 [−1.35 to −0.08]	−0.42 [−1.22 to −0.19]	*0.275*	*0.275*	*0.193*
Z-score LVIDs	−0.15 [−1.35–0.50]	−1.72 [−2.81 to −1.29]	−0.79 [−0.99 to −0.10]	−0.62 [−1.68–0.13]	***0.02***	*0.846*	*0.131*
RWT	0.39 [0.33–0.46]	0.46 [0.38–0.54]	0.39 [0.35–0.41]	0.36 [0.32–0.38]	*0.322*	*0.492*	*0.193*
LVM (g)	68.0 [48.7–87.6]	67.9 [47.3–81.7]	62.3 [50.3–73.8]	69.5 [52.1–75.0]	*0.492*	*0.275*	*0.131*
LVMi (g/m^2.7^)	50.0 [41.2–57.5]	47.6 [42.5–55.9]	41.4 [37.3–42.6]	35.8 [30.2–43.4]	*0.557*	***0.01***	***0.004***
Change in LVMi compared to T0		−0.8 [−3.2–1.0]	−8.9 [−15.6 to −5.4]	−14.3 [−15.3 to −12.9]			
SVi (ml/m^2^)	32.92 [25.55–41.94]	29.46 [26.47–35.31]	32.78 [30.53–35.20]	31.18 [30.35–32.23]	*0.695*	*0.77*	*0.695*
GLS (%)	−18.89 [−19.66 to −18.07]	−19.67 [−21.51 to −17.86]	−20.68 [−22.42 to −18.90]	−19.99 [−22.31 to −18.54]	*0.492*	*0.131*	***0.004***
Change in GLS compared to T0		−1.46 [−2.74 to 2.01]	−1.10 [−2.82–0.11]	−1.25 [−1.76 to −0.89]	*-*	*-*	*-*

Values are expressed as median with interquartile ranges. For comparison of T0 to T1, T2 and T3, the paired Wilcoxon test was used. Significant p-scores are in bold

*CI* cardiac index, *LV EF* left ventricular ejection fraction, *FS* fractional shortening, *IVSd* intraventricular septum thickness at end-diastole, *LVPWd* left ventricular posterior wall thickness at end-diastole, *LVIDd* left ventricular internal dimension at end-diastole, *LVIDs* left ventricular internal dimension at end-systole, *RWT* relative wall thickness, *LVM* left ventricular mass, *LVMi* left ventricular mass index, *SVi* stroke volume index, *GLS* global longitudinal strain

## Discussion

This study illustrates beneficial functional cardiac adaptation and reverse remodeling after pediatric kidney transplantation with large donor–recipient size discrepancy. We observed significant improvements in GLS within 1 year and a significant reduction in LVMi after 6 months, with further reduction at 1 year post-transplantation.

Prior to transplantation, most of the children in this cohort showed some degree of impaired strain, indicating subclinical myocardial LV dysfunction. Global longitudinal strain describes longitudinal systolic shortening of the left ventricle in a negative percentage value. It is a robust parameter allowing for detection of subtle changes in systolic LV function. Based on Levy et al.’s meta-analysis, GLS of smaller than −20.5% was considered impaired. 8/10 (80%) children met this criterion prior to transplantation. Notably, none of the children showed any clinical signs of cardiac dysfunction prior to transplantation. These findings are in line with earlier studies reporting dysfunctional GLS in, though typically older, pediatric patients with kidney failure and patients receiving hemodialysis [1, 7].

In our cohort, we found significant improvement in GLS 1 year after transplantation, with a mean improvement of −1.8% GLS, suggesting a recovery of systolic LV function. This is consistent with the findings from recent reports. Recently, Jain et al. showed a GLS improvement of −1.4% in adults who underwent living donor KT after a median follow-up of 239 days [6]. In a much larger adult cohort (*n* = 488) with a mix of living and cadaveric donors, Kim et al. showed varying GLS improvement, where lower GLS pre-transplant gave rise to more significant improvement afterwards [16]. Rumman et al. reported only significant improvement in GLS after transplantation in children who had previously received hemodialysis, though their cohort included a mixed cohort of living and cadaveric donor recipients and was older with a mean age of 11.5 years [17]. Xiao et al. found no significant change in GLS after 1 month in children receiving cadaveric donor transplants at a mean age of 10.6 years [7]. It is probable they did not find a functional improvement due to their short follow-up. In contrast to these studies, our study includes a longitudinal follow-up in young children transplanted with a relatively large, living donor kidney. In our follow-up, only 2 out of the 8 children with impaired GLS prior to transplantation showed recovery above the impaired GLS threshold of −20.5%. Extended longer follow-up is needed to determine whether normalization of GLS is achievable for all patients. However, the consistent improvement of GLS in this cohort supports the hypothesis that early transplantation, at young age, improves functional cardiac recovery, despite the hemodynamic challenge that the relatively large donor kidney imposes on the recipient.

Left ventricular hypertrophy is the most typical structural manifestation of kidney failure-associated cardiac remodeling [18]. As an indicator of hypertrophy, LVMi is an important prognostic parameter for cardiovascular-related mortality [18]. In this study, a significant decrease in LVMi was observed at 6 months after transplantation with further reduction after 1 year with a mean reduction of LVMi of 5.6 g/m^2.7^ and 12.7 g/m^2.7^, respectively. These results strongly suggest effective reverse remodeling. In pediatric KT, LV geometry has been more extensively studied than speckle-tracking echocardiography. A multitude of pediatric studies show varying improvements in LV mass or dimensions, although these cohorts generally include older children and a mix of cadaveric and living donors [17, 19–22]. Masuda et al. demonstrated significant LVMi improvement within 1 year in a small cohort of living donor recipients. Particularly, patients with LVH prior to transplantation showed similar decrease in LVMi to our patients [19]. Ramoglu et al. also observed reductions in LVMi similar to the present study within 1 year in cadaveric donor recipients. Rumman et al. showed a smaller degree of LVH regression in transplant recipients, though they also noted lower initial LVMi prior to transplantation [17]. Sgambat et al. reported progressively lower rates of abnormal LV geometry over a 42-month follow-up period. Recent studies in adults have also shown a significant decrease in LVMi after kidney transplantation [6, 16]. Normalization of blood pressure control is known to be a driving factor in LVH regression after pediatric kidney transplantation [22]. In our cohort, systolic blood pressure decreased significantly within 12 months after transplantation. Additionally, the proportion of patients on antihypertensive medication was 7/10 (70%) prior to transplantation and 3/10 (30%) 12 months after transplantation. These results support the notion that improved blood pressure significantly contributed to the observed improvement in LVMi.

The cardiorenal interplay that causes LVH and dysfunction as reflected in GLS is multifactorial and complex. Our study shows that cardiac recovery occurs in the context of a significant hemodynamic change induced by the relatively large donor kidney. As expected, at T1 in the PICU, CI was markedly elevated. The CI returned to pre-transplantation values at 1 year post-transplantation. Reduction of CI values and a proportional reduction in donor kidney size at 6 months post-transplantation has been reported in one study [23]. Up to now, a longer follow-up of CI has not been reported. Based on our results, we conclude that the initial hemodynamic challenge posed by a large graft does not hinder cardiac recovery; when managed appropriately, it may even facilitate excellent cardiovascular outcomes in pediatric recipients. In addition to excellent graft survival, renal function and catch-up physical growth, evidence of improved cardiac function further adds up to favor kidney transplantation in the very young, even with a relatively large donor [9, 24].

This study has several limitations. First of all, this was a single-center study with a small sample size, which limits the statistical power of our analyses and precludes any subgroup analyses. As such, our findings should be considered hypothesis generating. Furthermore, we focused our echocardiographic study on systolic function and LV geometry. STE-derived strain provided a sensitive marker for systolic function. However, diastolic function, an important marker for early cardiac impairment, was not evaluated. Lastly, it should be noted that the T1 measurement at ICU posed echocardiographic challenges: images were acquired in supine position, the interval between the surgery and exam varied and some patients still received inotropic support. Despite these limitations, the longitudinal prospective design and use of advanced STE strengthen the validity of our study.

## Conclusion

In pediatric kidney transplant recipients with large donor–recipient size discrepancy, LV global longitudinal strain improved within 1 year after transplantation and LV mass decreased within 6 months post-transplant. Together, these findings indicate that pediatric recipients can effectively adapt to the hemodynamic demands of a large graft and show improved LV function and reverse cardiac remodeling occurring as early as 6 months after transplantation.

## Supplementary Information

Below is the link to the electronic supplementary material.

Graphical Abstract
(PPTX 85.6 KB)

## Data Availability

The datasets generated during and/or analyzed during the current study are available from the corresponding author on reasonable request.
